# Investigating a mental effort explanation of the generation effect using pupillometry

**DOI:** 10.3758/s13421-025-01791-0

**Published:** 2025-09-04

**Authors:** Ania M. Grudzien, Nash Unsworth

**Affiliations:** Department of Psychology, University of Oregon, Eugene, OR 97403 USA

**Keywords:** Memory, Pupillometry, Generation, Attention, Mental effort

## Abstract

The “generation effect” is a phenomenon whereby people have better memory for information that is self-generated compared to information that is passively read. Throughout the years many theories have been proposed to explain this effect, one of which is the “mental effort theory,” which suggests that more mental effort is allocated to self-generated information, meaning that the act of generating information inherently requires more mental effort than processing existing information. In a series of four paired-associates memory experiments, pupillometry (an independent measure of effort) was used to investigate a mental effort explanation of the generation effect within-subjects, between-subjects, and in a third experiment, within-subjects while manipulating generation difficulty. In a fourth, follow-up experiment, a verbal component was added to draw a link between generation quality and the pupillary response. All four experiments showed that more mental effort was allocated to generated information compared to read information, and that this was accompanied by a boost in memory performance when performed within-subjects. Importantly, in a cross-experimental covariance analysis for all within-subjects experiments, we found that differential effort allocation partially accounts for the behavioral generation effect. Taken together, the pupillometry results lend support to the idea that a mental effort is associated with the generation effect.

## Introduction

In which case would you be more likely to remember a biology lecture on photosynthesis, a brand-new concept for you: if you re-read your notes, or if you generated a pictorial diagram of the process from start to finish? The growing body of literature in learning and memory has uncovered various memory strategies to enhance learning and subsequent recall. These strategies can be useful when trying to remember your grocery shopping list, a new route to the library, or new material learned in the classroom. Using the biology lecture example, according to the body of research on a memory strategy referred to as the “generation effect,” you would likely have better memory for the photosynthesis content if you drew your own diagram representing the information. Intuitively, the generation effect refers to the phenomenon whereby people have enhanced memory for information that is self-generated than for information that is passively read (McCurdy et al., 2020).

This effect was first described as “generation” by Slamecka and Graf (1978), who across five experiments robustly demonstrated that subjects had better memory for words that were generated based on a given stimulus and an encoding rule than if the words were simply read. This effect was found within-subjects, between-subjects, during self-paced and experimenter-paced trials, using free and cued recall memory tests. Later that year, Jacoby (1978) found a similar result pointing to the generation effect, namely that when participants play an active role in solving a crossword paired associate (PA) word unscrambling problem, they have better memory for the solution compared to if they are simply given the solution. The rationale provided by Jacoby, was that solving a problem requires an additional input of “consciousness” and being given the solution is, in contrast, effortless. When the difficulty of the paired associate (PA) word scrambling problem was increased by removing letters and replacing them with blanks, participants were expected to invest more effort in finding a solution, which should result in better memory. However, this was not the case. Participants did not have better memory for the more difficult, missing letter condition. These early studies opened the door for further scientific conjecture. From the time Slamecka and Graf first described this phenomenon in 1978, the generation effect has been reliably replicated for decades, and the role of effort in producing enhanced memory became a line of inquiry for other researchers.

### Generation and the mental effort theory

As evidence for the generation effect grew, various theories emerged in an attempt to explain it, yet little consensus has been reached based on any of them. The multiple theories can be organized into two groups: context memory theories, and item memory theories. Context memory theories address memory for the contextual details used to generate the memory item. Item memory theories address memory for the generated item itself (McCurdy et al., 2020). We begin our brief review of these two groups of theories by discussing the context memory theories. The “associative strengthening theory” is a context memory theory, and it suggests that the act of generation leads to greater processing of context clues, leading to better memory for those contextual details (Greenwald & Johnson, 1989). The “item-context trade-off theory” posits that generation requires more mental work, preventing one from processing unnecessary related contextual details, leading to better memory (Nieznański, 2012). A “processing account theory” suggests that the act of generation requires processing of context details, and the act of reading requires processing of perceptual details (Mulligan, 2004, 2011).

Next, we discuss the item memory theories. The “selective displaced rehearsal theory” suggests that participants rehearse generated words more compared to read ones, leading to better memory (McElroy & Slamecka, 1982). Another item memory theory, the “semantic activation theory” states that the generation effect occurs because generated stimuli are more meaningful. This theory predicts that when stimuli are not meaningful (e.g., nonsense words) the act of generation would not result in a memory boost. The “two-factor theory” suggests that generation allows for greater item-specific processing and relational processing resulting in better recall (Hirshman & Bjork, 1988). The item memory theory we investigate, originally alluded to by Slamecka and Graf and tested by Jacoby, is the “mental effort theory.” The “mental effort theory” proposes that more mental effort (i.e., attentional resources) are allocated to self-generated items at encoding, resulting in better memory at recall, given that more effort and attentional resources generally facilitate the formation of a stronger memory (McCurdy et al., 2020). McFarland et al. (1980) critiqued both Jacoby and Slamecka and Graff suggesting that the control condition, in which participants simply read the to-be-remembered information, was not sufficient to represent low effort, automatic processing. As stated by Slamecka and Graf, the generation effect could be attributed to the fact that self-generated information involves processing semantic meaning and is therefore less a measure of effort and more a method to force a deeper level of processing (Craik & Lockhart, 1972).

McFarland et al. (1980) addressed this issue by utilizing a task that required participants to either generate a word that rhymed with a cue word or to determine if an experimenter-provided word rhymed with a previously shown word. They also completed this version of the task with a semantic rule, by generating a word that would fit the context of a sentence or determine if an experimenter-given word fit the sentence. The idea was that differences in memory performance on the self-generated versus experimenter-provided terms would not be due to differences in level of processing, such that the control condition (decision task) still required effortful processing of the word in relation to its semantic or phonemic context. Free-recall results showed improved memory performance for self-generated words versus experimenter-provided words for both semantic and phonemic conditions. Thus, McFarland, Frey, and Rhodes argued that the generation effect observed here is not due to differences in effort or level of processing. It occurs due to the act of generating information which is, in itself, memorable, and independent of processing depth and type of encoding.

A potential explanation for the generation effect related to a mental effort theory is that the contrast of a generative (active) and passive version of the task and the resulting memory benefit is due to “selective displaced rehearsal,” such that the generative version requires more attentional resources at the cost of the easier, more passive read version (Fiedler et al., 1992). In other words, attention is directed to the more difficult generative version of the task (at the cost of the easier version), leading to the formation of a stronger memory. Taconnat and Isingrini (2004) similarly suggest that the effort hypothesis is predicated on the idea that greater mobilization of attentional resources to one condition allows for the memory boost. Several studies have investigated a mental effort explanation of the generation effect. They mostly use behavioral measures to quantify effort via response time on a dual-task. These dual-task experiments examine whether more attention and cognitive operations demanded by the primary task (task A), result in a longer reaction time for the secondary task (task B). Tyler et al. (1979) manipulated effort difficulty in a series of anagram-solving and sentence-completion tasks (the primary task A). They found that participants had better memory recall when the difficulty of the task was increased. Difficulty was manipulated based on whether generating a correct answer was intuitive given the context of the sentence. This was replicated in a second experiment, this time adding a secondary tone-detection task (task B) as a measure of mental effort. Response times were longer on the secondary task (task B) when the primary task (task A) was more difficult and required greater effort. However, when reaction times were corrected for reading time, response time for the various effort levels did not differ. In a follow-up experiment by the same authors, researchers found that when including a secondary tone-detection task, reaction times to the secondary task were longer for the high-effort anagram and sentence-completion tasks. These results suggest that when experimenters manipulated only the level of difficulty, and therefore only effort, the manipulation resulted in differential attention allocation. This leads to the conclusion that more difficult tasks are remembered better.

Other studies using similar anagram memory paradigms to manipulate effort find conflicting results. Foley et al. (1989) found that memory recall was superior for word solutions to easy anagrams compared to word solutions to difficult anagrams and words given by the experimenters. This result is not in line with a mental effort theory which suggests that more effort invested in solving difficult anagrams should result in better memory. It is worth noting that this paradigm did not have a task-independent measure of effort. Instead, reaction time for solving the anagram was the proxy for mental effort.

Hertel (1989) also tested the idea of whether the generation effect is linked to differences in mental effort. Using a similar anagram and sentence completion task, Hertel gave participants orienting tasks including generating solutions, verifying solutions, and evaluating solutions. The generating and verifying conditions both required participants to relate solutions to the given context, but the generating condition required a more active role in producing the solution based on a given completion rule. The evaluating task was designed to be the more effortful version of generating, in which participants would read the solution, see the context of the word problem, and decide which rule was used to generate the solution. Further, the difficulty of the problems within each orienting task condition (generating, verifying, evaluating) was manipulated. For anagrams, this was accomplished by changing the number of letters in the word that were rearranged. For sentence completions, this was done by manipulating the probability of generating the correct word to complete the sentence. It is critical to notice that the studies examining a mental effort explanation all manipulate difficulty differently, even when using similar tasks, like anagram solving.

Continuing with the prevailing research appoach, Hertel (1989) operationalized mental effort as response-time latency to a secondary tone-detection task, again borrowing from the idea that when more effort is expended, more resources are mobilized to the primary task, resulting in performance decrements in the secondary task. Their experiment results showed that generated solutions, for both anagrams and sentence completion, had superior recall compared to verifying solutions, and that verifying and evaluating tasks did not show any behavioral memory differences. Further, more difficult trials resulted in better memory, and this effect was greater for sentence completion. Analysis of response times on the tone detection task showed that latency varied across orienting tasks. The evaluating condition tone-detection latencies were longer compared to the verifying and generating conditions, and the generating-condition latencies did not differ from the verifying-condition latencies. Also, more difficult trials resulted in larger latencies for most orienting tasks (except verifying anagrams). This result is interesting, because although the generating condition provided better memory for solutions on a later free-recall task, researchers did not observe greater latencies to the tone detection task. The authors argue that cognitive effort, represented by the various orienting tasks, was not responsible for better memory performance because effort needs to be allocated to the memory item specifically and not the information used to produce it. Therefore, they cast their vote in support of an item memory theory of the generation effect as opposed to a context memory account.

Mixed results have also been observed when a generation manipulation has been applied within subjects versus between subjects. Generally, a larger generation effect is observed within-subjects and, often, between-subjects’ manipulations either diminish or abolish the effect all together (Bertsch et al., 2007). A potential explanation relates back to a selective displaced rehearsal account, that switching one’s attention between easy (read) and difficult (generate) versions of the task leads to better memory. This is to say that the generation effect is not due to the process of self-generating an answer, but due to the fact that more attention is focused on the stimuli that must be generated, and less on the stimuli that are read.

Given these mixed results, a recent meta-analysis examined the effect of condition type (read or generate) and generation difficulty (low and high) for all studies that included an effort manipulation and found that generated items were remembered better; however, there was no interaction, such that the magnitude of the generation effect did not depend on the difficulty level (McCurdy et al., 2020). As mentioned previously, the generation effect was larger when conducted within subjects (*M*_*diff*_ = 0.144, *p* < 0.001) than between subjects (*M*_*diff*_ = 0.073, *p* < 0.001), and a significant interaction was found comparing condition type (read vs. generate) and manipulation type (within vs. between) *F*(1, 119) = 34.27, *p* < 0.001. An earlier analysis suggested that effect sizes differ for within-subjects designs (*d* = 0.50) and between-subject designs (*d* = 0.28), and that an easier, partial word generation task (*d* = 0.32) has a smaller effect size than a more difficult generation task (*d* = 0.55) (Bertsch et al., 2007). However, these comparisons should be interpreted with very cautiously, considering that the data come from studies that use various manipulations of effort, some that are task independent, and some that are not.

### Pupillometry as a measure of mental effort

As demonstrated in this body of research, we lack a standardized technique to manipulate difficulty, and thus effort allocation. Another serious shortcoming of the studies discussed in this paper and in the McCurdy et al. (2020) meta-analysis is that they lack a measure of cognitive effort that is independent of the task itself. McCurdy et al. (2020) called for an independent measure of cognitive effort to solve this conundrum. Likewise, Kahneman (1973) proposed that to properly capture the involvement of mental effort, measures of mental effort should be independent of the cognitive task. Further, he provided a theoretical framework to solve this methodological problem; mental effort can be understood as attentional effort and can be operationalized by measuring sympathetic nervous system activity (Kahneman, 1973). He suggested that a viable means for tracking changes in sympathetic nervous system activity in the context of mental effort is through tracking changes in pupil dilation (Kahneman, 1973; for recent reviews, see also Beatty & Lucero-Wagoner, 2000; Goldinger & Papesh, 2012; Laeng et al., 2012; van der Wel, & van Steenbergen, 2018). This theoretical approach is also supported by an expanded model by Kanfer and Ackerman (1989) that provides a more updated theoretical framework of resource allocation, suggesting that cognitive performance is due to attentional capacity which differs between people and changes as a function of factors like attentional resource allocation across activities, perceived effort, external influences, self-regulation, motivation, etc.

A number of studies have utilized pupillary responses to examine encoding processes and mental effort (i.e., attentional effort) (e.g., Beatty & Lucero-Wagoner, 2000; Goldinger & Papesh, 2012; Heaver & Hutton, 2011; Janisse, 1977; Kafkas & Montaldi, 2011; Miller & Unsworth, 2020, 2021; Naber et al., 2013; Papesh & Goldinger, 2015; Unsworth & Miller, 2021a; Võ et al., 2007). For example, Taikh (2014) found that the pupil dilated more during encoding for deep processing tasks than for shallower processing tasks in a recognition memory task, suggesting that deep processing was more effortful than shallow processing (although see Gross & Dobbins, 2021). Similarly, Unsworth and Miller (2024) found larger pupillary responses for deep processed items than shallow processed items in a paired associate’s task, suggesting that more effort and attention resources were allocated for deep processing compared to shallow processing. Thus, if generation conditions require more mental effort (more attentional resources) than reading conditions, we expect that the pupil should dilate more for generation compared to reading, similar to what is seen with levels of processing manipulations.

#### Current study

To the extent of our knowledge, no studies to date have used independent, physiological measures of effort to test a mental effort explanation of the generation effect. Generally speaking, the size of the human pupil changes in response to numerous factors, such as light, emotional valence, and mental activity (Beatty & Lucero-Wagoner, 2000). Additionally, a great deal of prior research suggests that the pupil dilates in response to the amount of effort devoted to a task (for reviews, see Beatty & Lucero-Wagoner, 2000; Goldinger & Papesh, 2012; Laeng et al., 2012). These effects reflect task-evoked pupillary responses (TEPRs), where the pupil dilates relative to baseline levels due to increases in attentional effort across a number of tasks (Beatty & Lucero-Wagoner, 2000). In other words, a TEPR indexes the pupillary response to a stimulus upon its presentation. When a stimulus is presented, the pupil will dilate in response, especially when some attentional effort is needed. Upon reviewing the literature, Kahneman (1973) suggested that pupil dilation is a reliable and valid psychophysiological marker of attentional effort. That is, these task-evoked pupillary responses correspond to the intensive aspect of attention and provide an online indication of the intensity of attention (Just & Carpenter, 1993; Kahneman, 1973; Unsworth & Miller, 2021b).

The current study explored the generation effect using pupillometry and a paired associates (PA) paradigm which required memorization of associated word pairs for a later test. The design is like that of Hirshman and Bjork (1988), where participants were tasked with memorizing two lists of 14 word pairs, composed of a cue word and a target word which was the to-be-remembered item. In Hershman and Bjork’s design, half of the pairs belonged to the read condition, in which the cue and target were presented for the participants to read. Half of the pairs belonged to the generate condition in which vowels were removed from the target and replaced with blanks. Mirroring this design, in the current study we included two learning conditions, a read condition and a generate condition, where the read condition represents low effort, automatic processing, and the generate condition represents more effortful processing. This was followed by a cued recall test. This design also circumvents issues with previous research in that it places the to-be-remembered target item at the focus of attention for both learning conditions. A critique of previous literature was that in various anagram-solving tasks, the context of the memory item was at the focus of attention, instead of the to-be-remembered word. A PA task circumvents this issue, by placing the generated or read word at the focus of attention.

Our first aim was to replicate the generation effect using a within-subjects design, and to investigate whether differential amounts of effort are allocated to the two learning conditions. Our second aim was to conduct the same inquiry and replicate the generation effect behaviorally and physiologically between-subjects given the mixed results in past research. Our third aim was to vary generation difficulty by providing a low-effort generation condition and a high-effort generation condition and measure if effort is allocated differentially. The final aim was to link the dilation effect to the strength of association between the word generated and the word remembered. Our predictions, in line with the mental effort theory, were that participants would have better memory for the effortful generate condition and a greater pupil diameter on average compared to the read condition. We expected to replicate this result between-subjects. When generation difficulty was manipulated, we predicted that more effort would be allocated to the more difficult generation condition (evidenced by larger TEPRs) resulting in superior memory for the difficult generation pairs. Finally, we expected the generated words to be the same ones recalled at test, and that a potential pupillary effect would account for the variance in the behavioral generation effect. Note, prior research suggests that larger pupil dilations (and hence more attentional effort) are typically associated with better memory (e.g., Goldinger & Papesh, 2012; Miller & Unsworth, 2020; Unsworth & Miller, 2021a). However, it is not the case that there is a one-to-one mapping between pupil dilation and subsequent memory, as prior research has shown that in some conditions a larger pupillary response is actually associated with worse memory. For example, Miller et al., (2019; see also Unsworth & Miller, 2021a) found the largest pupillary dilations were associated with mid-list items on a delayed free-recall task. Consistent with classic serial position curves, these items tended to be recalled worse than primacy items. Unsworth and Miller (2021a) replicated these results and suggested they reflected cumulative rehearsal processing that built up (to a point) during encoding. Thus, greater effort was associated with these items given that participants were attempting to rehearse the items along with previously presented items, but these still tended to be recalled more poorly than primacy items. As such, differences in pupillary responses at encoding are driven by various factors, including amount of resources available and strategic allocation of those resources, which differ between people. In the current experiments we use pupillary responses to track the allocation of attentional effort during encoding.

## Experiment 1

Experiment 1 addressed the first aim, to replicate the generation effect within-subjects, and to measure physiological changes in allocated effort as indexed by changes in pupil dilation. We predicted that participants would have better memory for generated words and that this would be supported by an independent measure of effort, i.e., larger pupil dilations on average in the generate condition compared to the read condition.

### Method

#### Participants

Forty-one participants were recruited from the University of Oregon human subjects pool. Eighty percent of participants were female, 20% male. The average age of participants was 19 years (*SD* = 2.18). Participants were awarded course credit for their participation. Upon obtaining informed consent and demographic information, participants completed a PA cued-recall task. All participants were included in the final data analysis. We had a goal of getting a minimum of 35 participants per condition. With this sample size, we have sufficient power to detect medium to large effects (Cohen’s d) for within-subjects conditions.

#### Materials

Stimuli were composed of word pairs obtained from Nelson, McEvoy, and Schreiber’s Free Association Norms database (1998). Each word pair was composed of the cue word (presented on the left) and a target word (presented on the right). A total of 92 word-pair stimuli were produced by cycling through the alphabet, randomly choosing a cue word, and pairing it with its third associate, the target. In the Nelson database, each associated target word was given a level category based on the probability of freely generating that target word when presented with the cue. The third associate category was chosen based on the precedent set by Hirshman and Bjork (1988). Out of the 92 word pairs, 14 pairs were borrowed directly from Hirshman and Bjork (1988). After all the 92 pairs were compiled, the list was randomized. Then, all the vowels were removed and replaced with blanks (ex. “R_ _m” for “Room”) for the first half of the words in our list. These became the stimuli for the generate condition. The list was randomized again. From this randomized list, we created two practice rounds of five read, five generate words each, and three experimental rounds of 24 words each (12 read, 12 generate).

#### Procedure

The PA task was completed using a Tobii TX300 eye tracker to track changes in pupil diameter. Pupil diameter was simultaneously recorded binocularly at 120 Hz. To begin the data collection procedure, participants were seated in a dark room (illuminance ≈ 30 lx) in front of a computer with their heads affixed in a chinrest about 70 cm away from a computer screen measuring 20 × 11.5 in. Before receiving verbal and written instructions, participants completed an eye-tracker calibration procedure. To calibrate the eye tracker, participants were asked to fixate on a series of nine gray dots presented on a white background. All participants were successfully calibrated within the first few attempts.

**Fig. 1:**
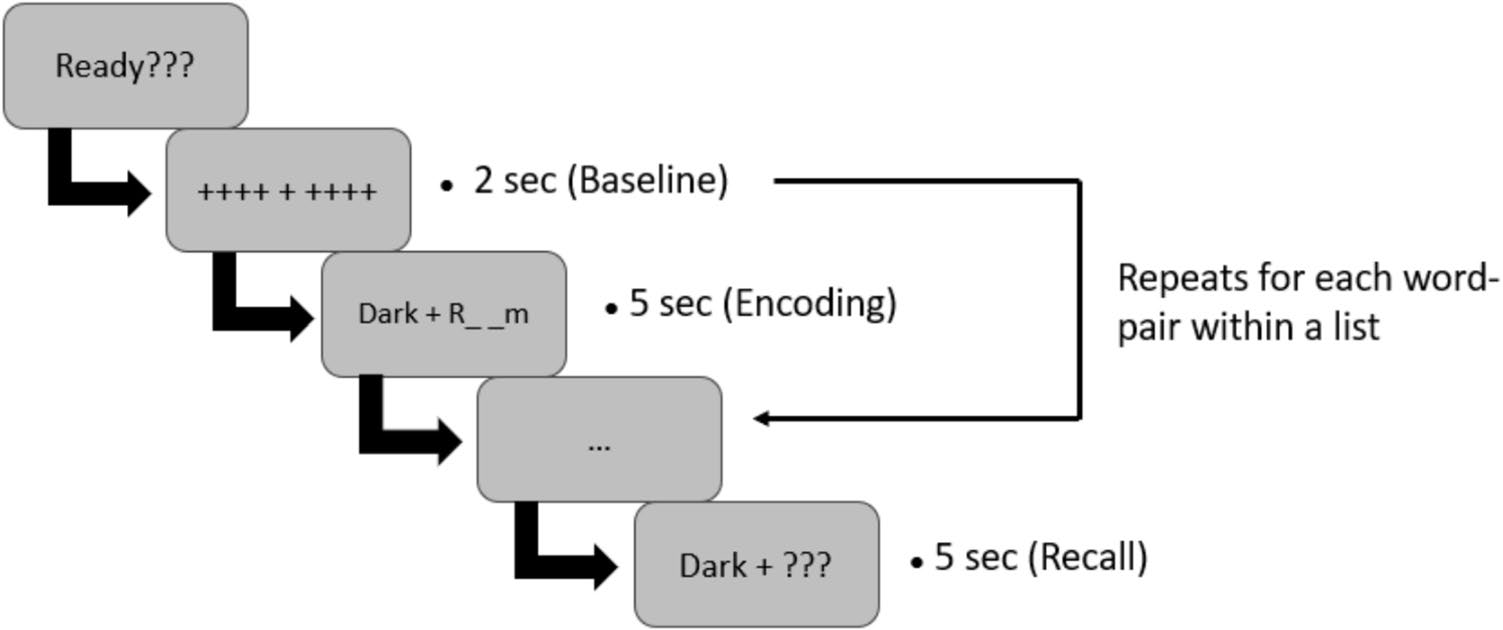
Experimental paradigm showing progression of encoding and recall phases

After obtaining verbal and written instructions, participants completed a typing exercise which required them to type a word presented on the screen. This was included to ensure that all participants were proficient typists. Then, two practice rounds of the PA task followed. Before each word pair was presented, a baseline pupil diameter measurement was obtained. The baseline measurement is the pupil measurement before each word pair is presented for encoding (see Fig. 1). Then, the pupil diameter fluctuates as the participant encodes each word pair. An experimental round consisted of an encoding phase and a test phase. During the encoding phase, we presented the entire word-pair list to participants. Participants were instructed to sit and observe, without interacting with the computer. A fixation cross was shown for 2,000 ms after which the word pair was displayed for 5,000 ms. After the presentation of the entire word-pair list came the recall, or test, phase. During the test phase, the cue word was displayed to prompt the participants to type in the target word. Participants had 5 s to type in the word, after which the next word was displayed. This experimental paradigm is illustrated in Fig. 1. This process was repeated for each practice and experimental round, although pupil measurements were only taken during experimental rounds.

#### Data analysis

##### Task-evoked pupillary responses (TEPRs)

In line with previous practices, data from the left eye was used to analyze the task-evoked pupillary response across all experiments. Pupil diameter was averaged across experimental rounds for each participant for each encoding time period (or “bin”), where a time bin represents a 200-ms section of the total encoding time (5,000 ms). Missing data points associated with eye-tracker malfunctions, blinks, or off-screen fixations were excluded from averaging (i.e., we did not interpolate for blinks and missing pupil data).

### Results

#### Behavioral results

Results from Experiment 1 addressed our first aim to replicate a behavioral generation effect and to investigate the role of mental effort using pupillometry. In line with this aim, we replicated a behavioral generation effect. We found that on average, participants had higher memory accuracy for generated words (*M* = 0.54, *SD* = 0.17) compared to read words (*M* = 0.49, *SD* = 0.20). A paired-samples t-test revealed this difference was statistically significant, *M*_*diff*_ = −0.046, *SD* = 0.12, *t*(40) = −2.55, *p* = 0.015. The effect size for this generation effect was substantial, *d* = 0.40.

##### TEPR results

**Fig. 2:**
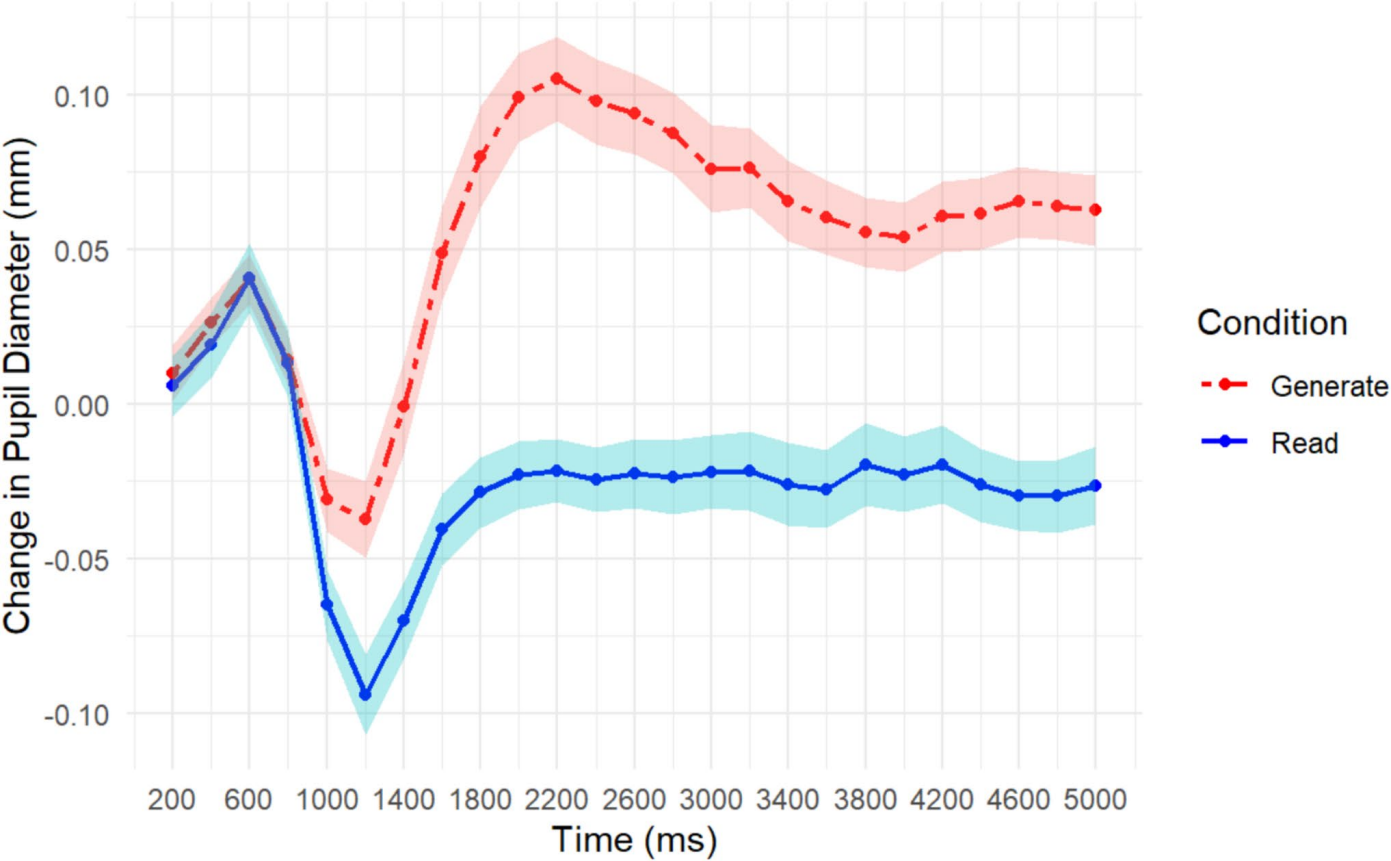
Change in pupil diameter from baseline across 5 s of encoding for each word-pair as a function of learning condition (read vs. generate). Shaded lines represent one standard error of the mean

To address the second part of our first aim, i.e., the involvement of mental effort, we analyzed the change in pupil diameter from baseline across the encoding period (5,000 ms). Ultimately, the pupillary results mirrored the behavioral results. A repeated-measures ANOVA showed a main effect of learning condition, *F*(1, 40) = 50.40, *MSE* = 0.061, *p* < 0.001, η_p_^2^ = 0.56, a main effect of encoding time period, *F*(24, 960) = 12.46, *MSE* = 0.005, *p* < 0.001, η_p_^2^ = 0.24, and an interaction *F*(24, 960) = 15.06, *MSE* = 0.002, *p* < 0.001, η_p_^2^ = 0.27. The main effect of learning condition suggests that pupil diameters were larger in the generate condition compared to the read condition. The main effect of encoding time suggests that across encoding the pupil dilated. The interaction shown in Fig. 2 suggests that participants’ TEPRs started at the same point and diverged from there; the generate word pairs diverged from baseline more compared to the read pairs. At the suggestion of a reviewer, we have also included additional analyses using corrected time bins which exclude the first five time bins in which the initial increase and drop in pupil diameter is due to the pupillary light reflex.^1^These results for each experiment can be found in the footnotes.

### Discussion

In Experiment 1, we found a significant behavioral effect of generation; we observed that the generative version of the PA task enhanced subsequent recall. Importantly, we found that pupil diameters were significantly larger in the generate condition, suggesting that participants allocated more effort to the words with missing vowels. This key finding is consistent with a mental effort theory, suggesting that increased mental effort (i.e., attention allocation) in generative learning is accompanied by a memory benefit.

## Experiment 2

Past research has shown mixed results in replicating the generation effect between-subjects. Often, applying learning conditions between subjects led to a decrease of the generation effect, which some posit is because differential allocation of attention does not occur due to lack of a contrasting, more effortful, condition which commands more attentional resources (Fiedler et al., 1992). Experiment 2 sought to test this idea and replicate the generation effect between subjects. We expected that more mental effort would be allocated in the generate group, and we also expected this to result in a memory benefit. Considering that generating information requires more cognitive operations, this should result in better memory for that information.

### Method

#### Participants

For a between-subjects design, we had a goal of obtaining a minimum of 35 participants per condition. With this sample size, we had sufficient power to detect medium to large effects (Cohen’s d). There were ultimately 41 participants in each learning condition, for a total of 82 participants from the University of Oregon human subjects pool. Participants’ average age was 19 years (*SD* = 1.12) and 70% of the sample was female, 30% was male. Participants were awarded course credit for their participation. Upon obtaining informed consent and demographic information, participants completed the PA cued recall task. All participants were included in the final data analysis.

#### Materials

Materials were the same as in Experiment 1, except that the learning condition manipulation was conducted between subjects, such that all words were either in the read condition or all were in the generate condition (i.e., all target words had missing vowels). The order of stimuli presentation was the same as in Experiment 1.

#### Procedure

The procedure for the second experiment was identical to the first. Changes in pupil diameter were tracked across encoding, as was memory accuracy.

### Results

#### Behavioral results

Results from Experiment 2 examining the generation effect between subjects failed to replicate a behavioral generation effect. When the learning conditions were applied between groups (each *N* = 41), the average memory accuracy did not differ significantly between the two learning groups, read *M* = 0.56, *SD* = 0.19 and generate *M* = 0.56, *SD* = 0.16. An independent-samples t-test confirmed this, *t*(80) = −0.018, *p* = 0.99. The effect size was also small, *d* = 0. A post hoc power analysis showed 1-*β* = 0.05. This suggests that when participants only participate in a generative memorization task without the contrast of a lower effort reading memorization task, a memory benefit is not incurred.

#### TEPR results

**Fig. 3:**
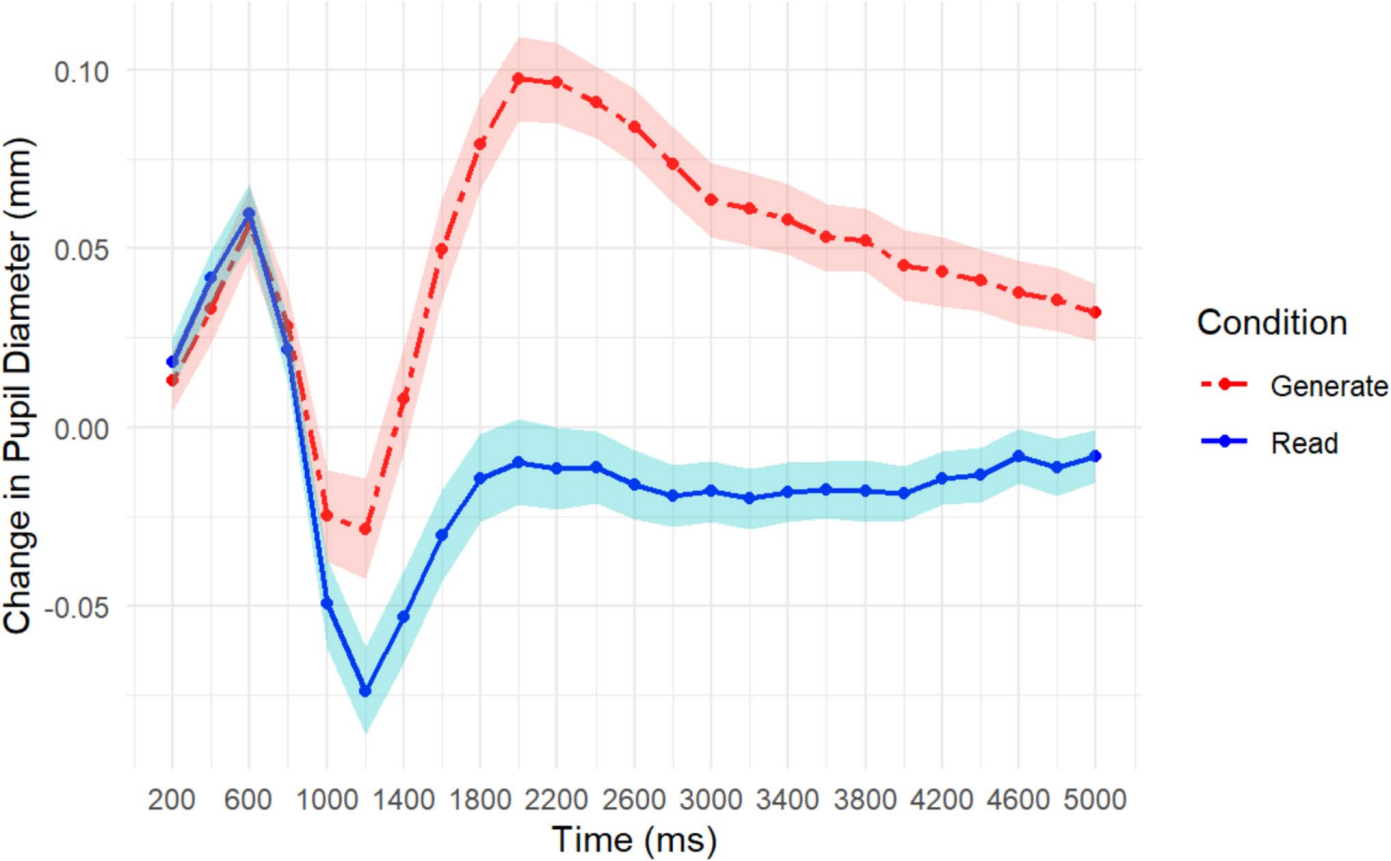
Change in pupil diameter from baseline across encoding for each word-pair as a function of learning condition (read vs. generate) conducted between subjects for Experiment 2. Shaded lines represent one standard error of the mean

The pupillary results suggest, however, that participants allocated differential amounts of mental effort in the two learning conditions. A repeated-measures ANOVA indicated a main effect of encoding period *F*(24, 1920) = 21.76, *MSE* = 0.002, *p* < 0.001, η_p_^2^ = 0.21, learning condition *F*(1, 80) = 34.91, *MSE* = 0.052, *p* < 0.001, η_p_^2^ = 0.30, and a significant interaction of encoding period and learning condition *F*(24, 1920) = 11.62, *p* < 0.001, η_p_^2^ = 0.13. As shown in Fig. 3, the pupil dilation effect was greater for the generate learning condition across the encoding period. This suggests that although participants in the generate group did not exhibit enhanced memory, they invested a great deal more effort to the task, overall, compared to the read group.

### Discussion

When the same paradigm was applied between-subjects, we did not find a behavioral generation effect. This is in line with the McCurdy et al. (2020) meta-analysis, which found that the magnitude of the generation effect decreases when learning conditions are applied between-subjects, and in some cases, is eliminated altogether. We did, however, find a large pupillary effect, suggesting that participants in the generate group allocated more mental effort to the word pairs overall compared to the read group. What is puzzling about this result, is that the increased mental effort did not pay off in terms of increased memory performance, showing that participants in the generate group invested effort without a behavioral payoff. This suggests that maybe it is the differential allocation *within* the context of a task that leads to better memory performance, such that the contrast between high- and low-effort processing may be a necessary condition to replicate this effect.

## Experiment 3

Experiment 3 addressed the third aim, to determine whether differential levels of effort are allocated to an easier generation condition, compared to a more difficult one. This experiment was conducted within-subjects. We expected that the difficult generation condition would result in a higher memory accuracy and larger TEPRs compared to the easy generation condition and read condition since the most mental effort should be invested to generate a more nuanced, third associate target word.

### Method

#### Participants

Fifty participants were recruited from the University of Oregon human subjects pool, all of which were included in the final data analysis. The mean age of participants was 19 years (*SD* = 1.14), and the sample was again 70% female and 30% male. Upon obtaining informed consent and demographic information, participants completed the PA cued-recall task. Participants were awarded course credit for their participation.

#### Materials

Materials from Experiment 1 were mostly reused, with some small exceptions. For each of the three experimental lists, two read and two generate pairs were removed (randomly), and ten new low difficulty generation pairs were added. These new generation pairs were composed of a new cue word and its *first* associate (target). Probabilistically, the first associate is most likely to be freely generated when prompted with its cue. Thus, the final stimuli set for Experiment 3 consisted of ten difficult generation pairs, ten easy generation pairs, and ten read pairs for a total of 30 word pairs per experimental list. An example of easy word pairs was accept + r_j_ct where the correct word to be generated is “reject.” A difficult generation pair for example was cabin + c_v_rn where the correct word to be generated is “cavern.”

#### Procedure

The procedure for the third experiment was identical to the first. Both changes in pupil diameter were tracked across encoding, as was memory accuracy.

### Results

#### Behavioral results

In Experiment 3, we varied generation difficulty across two levels (easy and difficult) within-subjects (*N* = 50) and found that memory accuracy differed across all conditions. A repeated-measures ANOVA showed a significant main effect of learning condition, *F*(2, 98) = 28.35, *MSE* = 0.010, *p* < 0.001, η_p_^2^ = 0.37. The read condition had the lowest accuracy (*M* = 0.43, *SD* = 0.19), followed by the difficult generation condition (*M* = 0.50, *SD* = 0.13). Lastly, the highest memory accuracy was observed for the easy generation condition (*M* = 0.58, *SD* = 0.15). An uncorrected post hoc t-test confirmed that the two generation conditions significantly differed from each other *M*_*diff*_ = 0.082, *t*(49) = 5.09, *p* < 0.001, although this difference is in the opposite direction of our original prediction. We expected that the difficult generation condition would have the highest memory accuracy due to increased allocation of effort, but instead found that to be the case for the easy generation condition. The read condition also significantly differed from the easy generation condition, *M*_*diff*_ = 0.072, *t*(49) = 3.26, *p* = 0.002.

#### TEPR results

**Fig. 4:**
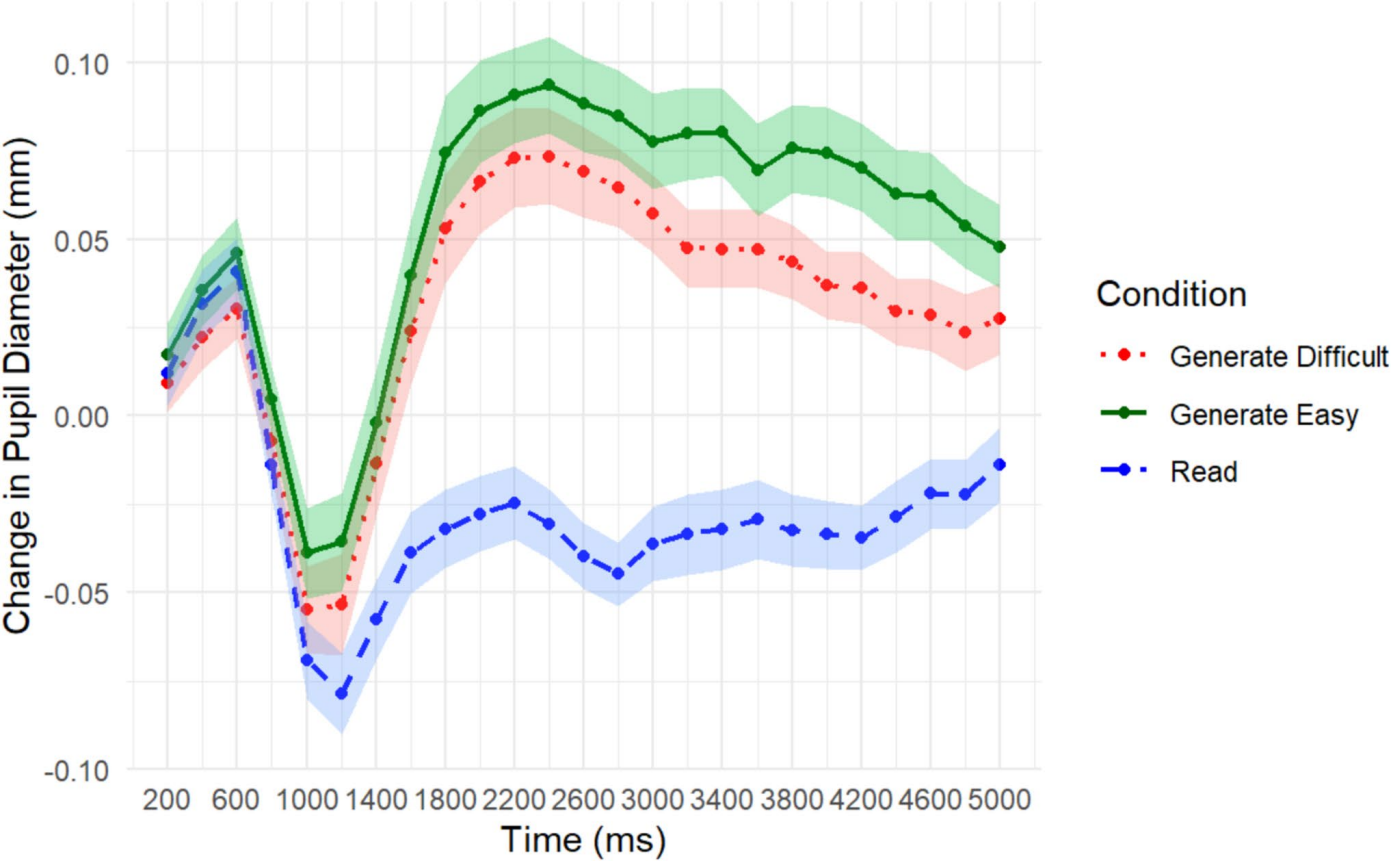
Change in pupil diameter from baseline across encoding for each word-pair as a function of learning condition (read vs. easy generation vs. difficult generation) for Experiment 3. Shaded lines represent one standard error of the mean

The pupillometry results present a similar pattern. Most notably, pupil size varied between all three learning conditions. A repeated-measures ANOVA yielded a main effect of learning condition *F*(2, 98) = 35.46, *MSE* = 0.062, *p* < 0.001, η_p_^2^ = 0.42, encoding period *F*(24, 1176) = 16.80, *MSE* = 0.007, *p* < 0.001, η_p_^2^ = 0.26, and interaction *F*(48, 2352) = 15.35, *MSE* = 0.002, *p* < 0.001, η_p_^2^ = 0.24. Critically, this interaction of learning condition and encoding time suggests that encoding time impacted the pupil size change in the read and generate conditions differently such that the three learning conditions started at baseline and diverged from there. A visual inspection of Fig. 4 suggests the largest difference in final pupil diameter was between the read condition and easy generation condition, easy generation having the largest pupil diameters.

To test if this difference was significant, we extracted the peak pupil diameter to conduct a pairwise comparison of pupil diameter between all conditions. At peak pupil dilation (2,200 ms into encoding) the pupil diameter in the read versus difficult generation condition significantly differed, *M*_*diff*_ = −0.10, *t*(49) = −6.94, *p* < 0.001, suggesting that greater pupil dilation occurred in the generation condition. Peak pupil diameter differed significantly between the read condition and the easy generation condition as well, *M*_*diff*_ = −0.12, *t*(49) = −8.17, *p* < 0.001, again showing that pupils were larger in the easy generation condition. Given our aim, we were most interested to see if there was a difference between the two generate conditions. A pairwise comparison found that the difference in peak pupil diameter in the two generation conditions approached significance, *M*_*diff*_ = −0.020, *t*(49) = −1.94, *p* = 0.058, *d* = 0.21. Post hoc power analysis showed that 1-*β* = 0.47. Because the two generation condition curves do not peak at exactly the same time, upon the suggestion of a reviewer, we also performed an individualized pupil peak analysis where we selected the peak pupil diameter for each participant and performed a paired-samples t-test. This test was statistically significant, *M*_*diff*_ = −0.030, *t*(49) = −3.04, *p* = 0.004, *d* = 0.43.

Post hoc, we decided to conduct a repeated-measures ANOVA (uncorrected) comparing only the difficult and easy generation conditions. This ANOVA yielded two significant main effects, a main effect of learning condition, *F*(1, 49) = 6.58, *MSE* = 0.047, *p* = 0.013, η_p_^2^ = 0.12, and a main effect of encoding time, *F*(24, 1176) = 23.33, *MSE* = 0.005, *p* < 0.001, η_p_^2^ = 0.32. The interaction was not significant *F*(24, 1176) = 1.24, *MSE* = 0.001, *p* = 0.20, η_p_^2^ = 0.025, with an observed power of 1-*β* = 0.92. We conducted this test because we noticed that the easy generation condition tended to have a more sustained dilation effect. These results suggest that when comparing the two difficulty conditions, pupils were larger in the easy generation condition.

### Discussion

In Experiment 3 we replicated a behavioral generation effect. We found statistically significant behavioral differences between the read and generate groups. We also found a statistically significant behavioral difference between the two generate groups (difficult and easy), yet the result was in the opposite direction predicted with the easy generation condition producing the best recall. There are at least two possible explainations. One possibility is that these results could be due to increased correct guessing of the target word in the easy condition at test, without correct generation at encoding (i.e. mind-blanking on the first try at encoding). Given that participants are most likely to freely generate the first associate target when prompted with the cue, probability is on their side. Thus, potentially due to the ease of association in the easy condition, the memory accuracy of the two generation conditions did not differ in the predicted direction. Additionally, the easy generation condition had a slightly larger pupillary response than the difficult generation condition. We predicted that the difficult generation condition would require additional mental effort evidenced by larger TEPRs and better memory; however, this prediction was not supported by the data. The question arises, why was there a large pupil dilation effect in the easy condition? As a reviewer suggested, part of the reason could be the contribution of other cognitive phenomena such as a target detection response or attentional orienting response to the target which would result in a dilation response (e.g., Preuschoff et al., 2011). That is, the peak dilation could reflect the amount of effort allocated to encoding processing, and the more sustained response seen at the tail end of encoding could reflect the additional contribution of an orienting response associated with generating the correct target response. Given the scope of the data, these alternative explanations could be contributing to the overall dilation effect and cannot be fully ruled out.

## Experiment 4

Experiment 4 was conducted to examine if participants were correctly generating words during the study phase (encoding) and recalling the words they generated (whether or not they generated the correct target) at recall. This experiment differed from the others in that it required participants to generate the target words verbally to address the link between generating, dilation, and the quality of associations at encoding and recall. We expected that participants would generate the correct target word at encoding, and recall it at a higher accuracy than the words that were simply read aloud. We expected to replicate the within-subjects pupillary effect found in Experiments 1 and 3.

### Method

#### Participants

Forty-six participants were recruited from the University of Oregon human subjects pool, all of which were included in the final data analysis. The mean age of participants was 19 years (*SD* = 1.12), and the sample was again 72% female and 28% male. Upon obtaining informed consent and demographic information, participants completed the PA cued recall task. Participants were awarded course credit for their participation.

#### Materials

Materials were identical to that of Experiment 1.

#### Procedure

The procedure for the fourth experiment was like that of the first, however, in Experiment 4 participants were instructed to verbalize both the cue and target word while a research assistant recorded their verbal responses. This ensured that the experimenter had insight into the words that participants were generating. Both changes in pupil diameter were tracked across encoding, as was memory accuracy.

#### Data analysis

In Experiment 4, we used two behavioral coding schemes. In the first, we marked a response correct if the recalled word matched the correct target word (i.e., the word they were supposed to generate). In the second coding scheme, we marked a response correct if it matched the word verbally generated at encoding, even if it was not the intended target word we had in mind. Task-evoked pupillary responses were analyzed in the same way as in Experiments 1 − 3.

### Results

#### Behavioral results

Results from Experiment 4 again replicated the behavioral generation effect. We observed that in the generation condition, 77% of the time participants generated the correct, experimenter-intended target word. Using coding scheme 1, we found that on average, participants had higher memory accuracy for generated words (*M* = 0.59, *SD* = 0.13), compared to read words (*M* = 0.51, *SD* = 0.18). A paired-samples t-test revealed this difference was statistically significant, *M*_*diff*_ = −0.085, *SD* = 0.14, *t*(45) = 4.02, *p* < 0.001*.* The effect size for this generation effect was large, *d* = 0.59. This effect did not change in significance when using coding scheme 2 (verbal responses). Generated words (*M* = 0.60, *SD* = 0.13) were remembered better compared to read words (*M* = 0.51, *SD* = 0.18). This means that 60% of the time, participants recalled the same word they themselves generated at encoding. A paired-samples t-test revealed this difference was statistically significant, *M*_*diff*_ = 0.095, *SD* = 0.14, *t*(45) = 4.50, *p* < 0.001.

#### TEPR results

**Fig. 5:**
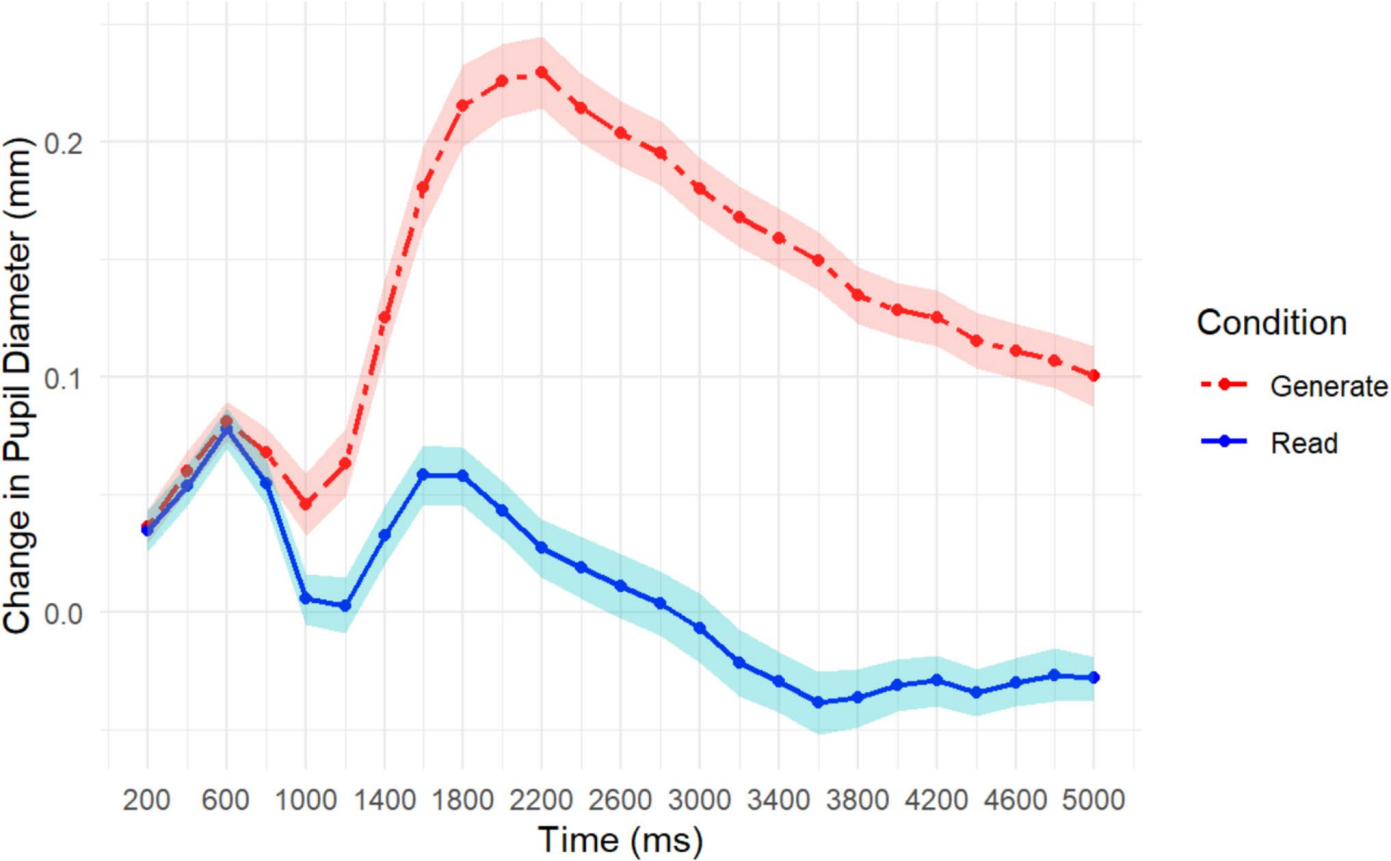
Change in pupil diameter from baseline across encoding for each word-pair as a function of learning condition (read vs. easy generation vs. difficult generation) for Experiment 4. Shaded lines represent one standard error of the mean

Replicating the results in Experiments 1 and 3, a repeated-measures ANOVA showed a main effect of learning condition, *F*(1, 45) = 199.53, *MSE* = 0.049, *p* < 0.001, η_p_^2^ = 0.82, a main effect of encoding time period *F*(24, 1080) = 18.54, *MSE* = 0.006, *p* < 0.001, η_p_^2^ = 0.29, and an interaction *F*(24, 1080) = 78.45, *MSE* = 0.001, *p* < 0.001, η_p_^2^ = 0.64. The main effect of learning condition again suggests that pupil diameters were larger in the generate condition compared to the read condition. The main effect of encoding time suggests that across encoding the pupil dilated. The interaction shown in Fig. 5 suggests that across encoding, pupil diameter diverged more from baseline for the generate condition compared to the read condition.

### Discussion

In Experiment 4 we replicated a behavioral generation effect and a pupil effect suggesting that mental effort is part of the generation process. Given that in Experiments 1 − 3 it was unclear if incorrect responses were reflective of true memory errors, we used a verbal generation paradigm in Experiment 4 and employed a new coding scheme, which allowed us to mark responses correct if that was what participants generated at encoding. This allowed us to connect the quality of the generated content to pupil dilation, the neural correlate of mental effort. We found that both coding schemes gave significant, and almost identical, behavioral results. Arround 60% of the time, participants generated and recalled the correct experimenter target word. Thus, the quality of generations was reliable and related to significant changes in pupil diameter. Here, we show evidence that during the generation process, participants successfully generate the target words, and that mental effort, as evidenced by the pupillary effect, is part of the generation process, although not entirely responsible for the behavioral memory effect.

## Cross-experimental analysis

Across all within-subjects experiments we observed a behavioral generation effect, as well as a pupil dilation effect. Given the somewhat ambiguous relationship between the pupil and behavioral generation effect, we ran a post hoc, exploratory analysis to assess the contribution of pupil differences in the behavioral differences between the two learning conditions in within-subjects Experiments 1, 3, and 4. For the purposes of equal comparison, we only used the difficult generation condition for Experiment 3, since it mirrored the same word pairs used in Experiments 1 and 4. The easy condition was composed of different word pairs (first associates) not used in Experiments 1 and 4. A strong hypothesis of the mental effort explanation to generation would predict that when the pupillary generation effect (indexing differences in effort allocation) is covaried out, the behavioral generation effect should no longer be significant. That is, effort is fully accounting for the generation effect. A weaker hypothesis is that effort only partially accounts for the generation effect. This hypothesis predicts that when the pupillary generation effect is partialed out, the magnitude of the generation effect should be reduced (although still significant). To examine this, we conducted a repeated-measures analysis of covariance on accuracy for the read and generate conditions and covaried out the pupillary generation effect (i.e., the difference in peak pupil diameter between the generate and read conditions). Table 1 shows the generation effects in Experiments 1, 3, and 4 before covarying out the pupillary responses and after covarying out the pupillary responses. As can be seen, in Experiments 1 and 4 when the pupillary responses were covaried out, the behavioral generation effect was no longer significant. In Experiment 3 it remained significant. Importantly, in all three experiments the magnitude of the generation effect (*ηp*^*2*^) reduced substantially, suggesting that a large portion of the variance in the behavioral generation effect was shared with the pupillary generation. We also examined this by combining the data in all three experiments and found that the behavioral generation effect remained significant, but the magnitude of the effect decreased. Collectively, these results are consistent with the weak hypothesis, suggesting that differential effort allocation partially accounts for the behavioral generation effect.

**Table 1:** Cross-experimental analysis covarying out the effect of pupil dilation

	Generation effect		Covarying out pupil	
	*p*	*ηp*^*2*^	*p*	*ηp*^*2*^
E1	0.015	0.14	0.76	0.019
E3	0.002	0.18	0.02	0.12
E4	< 0.001	0.27	0.09	0.07
Combined	< 0.001	0.19	0.002	0.07

## General discussion

In the current study, we tested a mental effort theory as an explanation of the generation effect. We set out to replicate the generation effect within and between-subjects, to track mental effort investment via an independent measure of effort, and to modulate the generative version of the task (providing two levels of effort allocation) and track effort allocation. In a series of experiments, we successfully replicated a generation effect within-subjects. We ran a follow-up experiment (Experiment 4) which gave us the insight that participants were able to successfully generate the intended experimenter target word the majority of the time. Across all four experiments, we consistently demonstrated that more mental effort (i.e., attention) was allocated to the more difficult generation condition as evidence by larger TEPRs, even when this did not result in a behavioral generation effect (between-subjects). We found this pupillometry effect within-subjects in Experiment 1, between-subjects in Experiment 2, and we replicated a within-subjects pupillometry effect in Experiments 3 and 4. Our pupillometry results are consistent with a mental effort theory, suggesting that mental effort is associated with generation.

Importantly, the cross-experimental analysis we conducted across all within-subjects experiments provided evidence for mental effort’s involvement in this memory phenomenon. Across all within-subjects experiments, the magnitude of the behavioral generation effect (*ηp*^*2*^) reduced substantially, suggesting that a large portion of the variance in the behavioral generation effect was shared with pupillary generation. This was also true for the combined dataset. Collectively, these results suggest that differential effort allocation (measured with pupil) partially accounts for the behavioral generation effect. This is a key finding, because it shows that mental effort, as captured by variation in pupil size, explains a substantial portion of the variation we observed in the behavioral generation effect. This lends support to the conclusion that the act of engaging in effortful word generation results in the formation of a stronger memory. This is not to say that mental effort is the only factor leading to better memory, but it seems to be an important contributor. We by no means propose a monolithic theory of generation, in that mental effort fully explains the effect. If this were the case, we would have expected a larger decrease in the behavioral generation effect when the pupillary generation effect was partialed out, which we did not observe. Nevertheless, evidence from this cross-experimental analysis shows that the contribution of mental effort in the behavioral generation effect is not trivial.

Our results were also not without some nuance. We did not replicate the generation effect behaviorally between-subjects, but we did find a large between-subjects physiological effect, suggesting that more effort was allocated to the more effortful generative version of the PA task. This points to the conclusion that differential mental effort allocation is a factor at play in the generation effect, suggesting potential support for selective displaced rehearsal. Another possible explanation for this result is that participants were laboring in vain, i.e., providing more mental effort without a performance pay-off. The “labor-in-vain effect,” first described by Nelson and Leonesio (1988) in the judgement-of-learning literature, refers to the case when additional time and effort is allocated to learning without an increase in correct recall. This labor-in-vain effect also points to the core concept that there is no perfect one-to-one relationship between effort investment and performance. This is akin to what we observed; the additional effort investment of participants in the generation condition did not always result in improved memory. Thus, pupillary responses can be used to track the allocation of attentional effort, but it is not necessarily the case that more effort (greater dilation) always results in better memory (e.g., Miller et al., 2019; Unsworth & Miller, 2021a).

The next puzzling result came from Experiment 3. We replicated the generation effect within-subjects but found that the easy generation condition had better recall than the difficult generation condition. As noted previously, this could be due to increased correct guessing at recall in the easy condition. Given that the first associate target word is easiest to associate when prompted with the cue, there is a high probability that participants could have generated the word correctly at test without generating it at encoding. Additionally, the easy generation condition tended to have a slightly larger and more sustained pupillary response than the difficult generation condition. As noted previously, this could reflect an additional contribution of an orienting response associated with generating the correct target response which is more likely to occur in the easy condition. Future research is needed to better examine this hypothesis.

Overall, these results suggest that mental effort, operationalized as pupil dilation, is at least partially responsible for the memory benefit incurred by the generation effect. In the cross-experimental analysis, when we covaried out the pupillary generation effect, the magnitude of the behavioral generation effect dropped across all three within-subjects experiments and the combined dataset. This suggests that mental effort is partially contributing to the behavioral generation effect. As we have alluded to, there are many other theories about why the effect occurs. The generation effect is a multi-faceted, complex process, and the evidence provided here points to the fact that a mental effort account is part, but not all, of the story. Some of the results we discussed point to a “selective displaced rehearsal theory,” meaning there may be multiple conditions that are necessary to replicate this effect.

### Limitations and future directions

It is prudent to consider possible limitations in the experimental design of this study. Most notably, our operationalization of the low effort condition in Experiment 3 presents a possible confound of increased guessing, leading to what, on paper, looks like better memory performance. To circumvent this, future research could employ a different design, where participants would verbally generate the target words out loud at encoding and again at test, allowing the researcher to check if the correct word is generated at encoding and recalled at test. We employed this technique in Experiment 4 and found that when using the third associates (difficult generation condition of Experiment 3) there was no difference in performance using the two coding schemes, one for verbal responses, one for the intended target word. Second, due to our task design, participants had only 5 s to type out responses, which often resulted in partially coherent and incomplete answers, leading to scoring inaccuracies. This could be remedied in the future by providing participants with more time in the recall phase.

Another statistical limitation is that the pupil time bins are autocorrelated in sequence, such that each value depends strongly on the immediately preceding one, due to the fact that the pupil cannot instantly change size. Because repeated-measures ANOVAs do not necessarily represent this kind of structured dependency, we have included a linear mixed-effects model with an autoregressive feature in footnote 2 for each experiment. The result of these linear mixed-effects models with the autoregressive feature which accounts for the autocorrelation in the pupil data supported the ANOVA results. The linear model showed that in most experiments the interactions between learning condition and encoding period were significant.^2^ This gives us confidence that after controlling for the natural similarity of adjacent pupil measurements, changes in pupil over time were still significantly different between trial types, with some nuance in Experiment 3, again confirming what we found in the ANOVA. Overall, the results of this additional analysis mirrored the main results (interactions) from the repeated-measures ANOVAs. Future studies employing a similar paradigm might consider improving upon these design quirks.

Given that we found significant behavioral and physiological evidence of the involvement of mental effort in the generation effect across all four experiments, several practical applications may be extracted. Future research may consider how within-subjects reading versus generating learning conditions might be applied in real-world learning settings like the classroom or workplace. As mentioned, it may be task switching between high- and low-effort conditions which provides the necessary contrast to incur a memory benefit. Therefore, including a mix of active generating activities and passive reading and listening activities may be effective in facilitating learning. Educators and researchers alike might consider this aspect when designing educational activities and future studies.

## Conclusions

Through a series of four experiments, we have provided evidence that mental effort is one possible explanation for why the generation effect results in a memory benefit. Across all four experiments, more mental effort was invested to generated stimuli versus read stimuli, and when learning conditions were applied within-subjects, this was accompanied by a memory benefit. In the fourth experiment, we demonstrated the strength of association between word pairs in the generation condition was related to the dilation effect. Further, a cross-experimental covariance analysis showed that when variance in the behavioral effect due to pupil dilation is partialed out, the effect substantially decreased. This suggests that mental effort, in part, contributes to memory accuracy. These results are consistent with a mental effort explanation of the generation effect, suggesting it may be in part caused by the fact that more attentional resources are allocated when generating words.

## Data Availability

Data and materials are available upon request. None of the studies were preregistered.

## Footnotes

1 Using corrected time bins which eliminate the pupillary light reflex, a repeated measures ANOVA for Experiment 1 showed a main effect of learning condition, *F*(1, 40) = 10.15, *MSE* = 0.058, *p* = 0.003, ηp2 = 0.20, a main effect of encoding time period *F*(19, 760) = 14.59, *MSE* = 0.004, *p* < 0.001, ηp2 = 0.27, and an interaction *F*(19, 760) = 4.68, *MSE* = 0.002, *p* < 0.001, ηp2 = 0.11. Similarly, Experiment 2 indicated a main effect of encoding period *F*(19, 1520) = 28.78, *MSE* = 0.001, *p* < 0.001, ηp2 = 0.27, learning condition *F*(1, 80) = 4.60, *MSE* = 0.073, *p* = 0.035, ηp2 = 0.054, and a significant interaction of encoding period and learning condition *F*(19, 1520) = 7.31, *p* < 0.001, ηp2 = 0.084. Experiment 3 also yielded a main effect of learning condition *F*(2, 98) = 25.92, *MSE* = 0.035, *p* < 0.001, ηp2 = 0.35, encoding period *F*(19, 931) = 24.50, *MSE* = 0.004, *p* < 0.001, ηp2 = 0.33, and interaction *F*(38, 1862) = 7.28, *MSE* = 0.001, *p* < 0.001, ηp2 = 0.13. Finally, Experiment 4 showed a main effect of learning condition, *F*(1, 45) = 110.89, *MSE* = 0.041, *p* < 0.001, ηp2 = 0.71, a main effect of encoding time period *F*(19, 855) = 28.57, *MSE* = 0.004, *p* < 0.001, ηp2 = 0.39, and an interaction *F*(19, 855) = 25.80, *MSE* = 0.001, *p* < 0.001, ηp2 = 0.36.

2 A linear mixed-effects model was used to examine the effects of learning condition, encoding period, and their interaction on pupil size. The model included a random intercept for subjects and an AR(1) correlation structure to account for autocorrelation across time bins. For experiment 1, results showed a significant main effect of encoding period, *b* = 0.0022, *SE* = 0.0007, *t*(2006) = 3.24, *p* = 0.0012. The main effect of learning condition was not significant (*p* = 0.22). However, the interaction between learning condition and encoding time was significant, b = 0.0034, *SE* = 0.001*, **t*(2006) = 3.45, *p* < 0.001, indicating that pupil response trajectories differed by condition. This significant interaction mirrors what was found in the repeated measures ANOVA. Experiment 2 did not show a significant main effect of encoding period (*p* = 0.15) or learning condition (*p* = 0.18). However, the interaction between learning condition and encoding time was significant, *b* = 0.0034, *SE* = 0.0019, *t*(1966) = 2.21, *p* = 0.027, indicating that pupil response trajectories differed by condition. This significant result was again consistent with our main findings. For experiment 3, we ran a single model with encoding period and learning condition (now three groups including read, easy generation, difficult generation) where the difficult generation condition was the reference group. This model found no significant main effects of learning condition or encoding period (*p* >.05). The interaction between learning condition and encoding period was significant only for the reading condition (*b* = -0.0019, *SE* = 0.00094, *t* = 2.09, *p* = 0.037), not for the easy generation condition (*p* = 0.51), where the difficult generation condition was the reference group. This suggests that the trajectory of pupil dilation over time differed for read pairs compared to the difficult generation condition, but this is not the case for the easy generation pairs. In other words, no significant difference in time trajectory was observed between easy and difficult generation pairs, suggesting similar patterns of pupil change over time in these two conditions. This interaction result is again consistent with what the repeated measures ANOVA found. Lastly, experiment 4 results showed a significant main effect of encoding period, *b* = 0.0022, *SE* = 0.0007, *t*(2251) = 3.53, *p* < 0.001. The main effect of learning condition was not significant (*p* = 0.22). However, the interaction between learning condition and encoding time was significant, *b* = 0.0054, *SE* = 0.001, *t*(2251) = 5.31, *p* < 0.001, indicating that pupil response trajectories again differed by condition, as we found earlier in the repeated measures ANOVA.

## Code availability

Available upon request.
